# Filiform fire needling therapy relieves T cells-mediated melanocyte apoptosis and dysfunction by inhibiting JAK/STAT3 pathway via Mfsd4a in vitiligo

**DOI:** 10.1186/s13020-025-01172-4

**Published:** 2025-07-24

**Authors:** Yue Shi, Dong Chen, Yao Wang, Cong Zhang, Yana Cao, Yan Liu, Ting Song, Cheng Tan, Yongjun Peng

**Affiliations:** 1 Dermatology Department, Affiliated Hospital of Nanjing University of Chinese Medicine, Nanjing, China; 2 Acupuncture and Rehabilitation Department, Affiliated Hospital of Nanjing University of Chinese Medicine, Nanjing, China; 3 Clinical Laboratory, Affiliated Hospital of Nanjing University of Chinese Medicine, Nanjing, China

**Keywords:** Filiform fire needling therapy, Vitiligo, Mfsd4a, Melanocytes, Melanin

## Abstract

We investigated the molecular mechanism of filiform fire needling therapy (FFN), an effective treatment option for vitiligo, focusing on its role in relieving depigmentation. Firstly, we validated the efficacy and safety of FFN in a study with 11 enrolled vitiligo patients. We then found that the depigmentation score was significantly improved in monobenzone-induced vitiligo mice treated with FFN. Subsequently, after being co-cultured with T-cells extracted from FFN-treated lesions, apoptosis of melanocytes was reduced and melanogenesis was enhanced. Furthermore, the gene Mfsd4a was significantly differentially expressed in melanocytes between the model group and the FFN intervention group. Further in *vitro* verification showed that JAK/STAT3 pathway activity was inhibited, and melanocyte activity was enhanced after knocking out Mfsd4a in co-cultured melanocytes from the monobenzone group. Moreover, interference with Mfsd4a increased MITF transcription, leading to TYR activation and promotion of melanin formation. Lastly, we found that IL-6 was involved in regulating Mfsd4a-mediated JAK/STAT3 pathway suppression, thereby regulating melanocyte survival and melanogenesis. These results demonstrate that FFN alleviates T cell-mediated melanocyte apoptosis and dysfunction by inhibiting the JAK/STAT3 pathway signaling pathway via Mfsd4a to treat vitiligo.

**Supplementary Information:**

The online version contains supplementary material available at 10.1186/s13020-025-01172-4.

## Introduction

Vitiligo, a common autoimmune dermatosis, is characterized by the destruction of melanocytes due to T cells-mediated melanocytes apoptosis and dysfunction, which results in well-defined depigmented patches on the skin and mucosa [1]. Although not a life-threatening disease, serious psychosocial consequences are undeniable [2, 3]. Previous studies have shown that vitiligo is mainly triggered by various factors such as abnormal immune responses, genetic susceptibility, oxidative stress, metabolic changes, and environmental factors, with short remission periods and high recurrence rates [4, 5]. Current treatment options for vitiligo are mostly generic, with limited efficacy and significant side effects, necessitating the need for effective natural therapies [6].

Acupuncture, as an indispensable part of Chinese Medicine, has been used to treat various diseases, including vitiligo [7]. Filiform fire needling therapy is a form of acupuncture originating from the "burning needle" mentioned in the *Huangdi Neijing*. Previous studies have evaluated the clinical efficacy and safety in patients with vitiligo [8–11], but the certain mechanism remains less understood so far.

In the current study, we found that the filiform fire needling therapy effectively relieved skin lesions in patients and mouse models of vitiligo. We further isolated T cells from the lesions with or without filiform fire needling therapy and co-cultured them with melanocytes to analyze the molecular mechanism of filiform fire needling therapy in vitiligo.

## Materials and methods

### Sample collection and treatment

We enrolled cases with non-segmental vitiligo who visited in the dermatology or acupuncture and rehabilitation outpatient clinics of the Affiliated Hospital of Nanjing University of Chinese Medicine (also named as Jiangsu Province Hospital of Chinese Medicine) from June 2023 to February 2024. Inclusion criteria defined as follows: (1) stable non-segmental vitiligo, (2) lesion area ≤ 10% Body Surface Area, and (3) without other treatment within the past 4 weeks. Exclusion criteria defined as follows: (1) lesion restricted on the face or mucous membranes; (2) with history of other immune disorders, coagulation diseases, severe cardiopulmonary diseases or inadequate liver or kidney functions; (3) participated in other clinical trials within recent one month.

Filiform fire needling therapy procedure was performed by the physician in the acupuncture rehabilitation department. Acupuncture needles of Huatuo brand with specification 0.3 mm × 25 mm were selected. After burning with an alcohol lamp, prick from the outer edge to the center of the lesion one by one, and finally prick surround the lesion with needles, retaining them for 30 min. The needle spacing should be 3–5 mm. The schedule was defined as once every two weeks (± 2 days) for 8 weeks. Their peripheral blood samples were collected when baseline and after treatment. Present study was approved by the Ethics Committee of Affiliated Hospital of Nanjing University of Chinese Medicine. All patients voluntarily participated in the study and signed informed consent forms.

### Animals

Four-week-old female C57BL/6 mice were obtained from Aniphe BioLab (China) and maintained under standard laboratory conditions. We first depilated the back of the mice using a 1:1 mixture of rosin and paraffin, with the test area about 2 cm × 2 cm. The control group applied 50 mg of vaseline to the test area once daily for 60 days. We induce the vitiligo model by applying 40% monobenzone, 50 mg, to the depilated area of the mice once daily for 60 days. Filiform fire needling therapy was initiated on the 31st day of monobenzone treatment at the same time, with the method being slightly tightening the depigmented skin with the left hand, holding the needle with the right hand, heating the filiform fire needle to red-hot over an alcohol lamp, and quickly and evenly puncturing the skin with an average spacing of 1.5 mm and depth to the superficial dermis every three days until day 60.

### Depigmentation scoring

After modeling, the depigmentation changes of the mice’s skin and hair in each group were observed and scored according to Mosenson et al. [12]. The scoring criteria were: 0, no depigmentation; 1, mild depigmentation (depigmentation area below 10%); 2, moderate depigmentation (depigmentation area between 10 and 25%); 3, significant depigmentation (depigmentation area between 25 and 50%); 4, severe depigmentation (depigmentation area between 50 and 75%); 5, very severe (depigmentation area above 75%).

### Cell culture and treatment

Mouse melanocyte melan-a was purchased from American Type Culture Collection (ATCC) and cultured in RPMI 1640 medium. For mouse T lymphocytes, skin lesion tissue was collected from mice before and after treatment, scraped off the surface, added with 5 mL of ficoll separation solution in a 15 mL centrifuge tube, and the PBS containing lymphocytes was slowly added to the centrifuge tube, centrifuged at 600 rpm for 25 min, and the middle layer liquid was extracted. After adding PBS and centrifuging at 600 rpm for 5 min, the precipitate was retained, added with 1 mL of red blood cell lysis solution for 5 min, centrifuged to retain the precipitate, and resuspended with lymphocyte culture medium.

Melanocytes were seeded in a 6-well plate at a density of 1 × 10^5 cells per well and cultured for 4 h in an incubator, then added with lymphocytes at a ratio of 1:5, co-cultured for 3 days, and then the supernatant and suspended lymphocytes were removed, retaining the melanocytes.

### Melanin content and secretion detection

Melanocytes were seeded in a 6-well plate at a density of 5 × 10^5 cells and incubated at 37 °C in a CO_2_ incubator. For melanin content, the cell number was adjusted to 1 × 10^5, NaOH solution was added, treated at − 80 °C for 4 h, and the absorbance was measured at 405 nm. Melanin content was calculated based on the treatment group/control group (RPMI treated) ratio. For melanin secretion, the melanocyte culture medium was centrifuged at 2000 rpm for 3 min, and the absorbance was measured at 405 nm, calculated similarly.

### ELISA

TNF-α (CB10851-Mu/CB11762-Hu, COIBO BIO), IL-1β (CB10173-Mu/CB10347-Hu, COIBO BIO), and IL-6 (CB10187-Mu/CB10373-Hu, COIBO BIO) levels in tissues and melanocytes were detected using ELISA kits according to the manufacturer’s instructions.

### RT-qPCR

Total RNA was extracted using Trizol (15596018CN, Invitrogen) and cDNA was synthesized using PrimeScript RT Reagent Kit (RR037Q, TAKARA) according to the manufacturer’s instructions. qRT-PCR was performed using SYBR green PCR Master Mix (QPK-201, TOYOBO) on the 7500 Real-Time PCR System (Applied Biosystems). Relative expression of target genes was calculated using the 2–ΔΔCT method.

### CCK8 cell viability assay

Cell viability was measured using the CCK8 kit (HY-K0301, MedChemExpress) according to the instructions. Briefly, cells were seeded in a 96-well plate, CCK-8 reagent was added to each well, incubated, and absorbance was read at 450 nm using a microplate reader.

### Colony formation assay

Melanocytes were seeded in a 6-well plate at a density of 500 cells per well. After 14 days of incubation, cells were fixed with 4% paraformaldehyde (E672002, Sangon Biotech) for 30 min and stained with 0.1% crystal violet solution (V5265, Sigma-Aldrich). Colony numbers were counted.

### Flow cytometry

Cells were seeded in a 6-well plate (1 × 10^6 cells per well) and digested with 0.25% trypsin (without EDTA), centrifuged at 300 g for 5 min at 4 °C, and washed with PBS. Samples were stained using the Annexin V-PE/7AAD kit (559763, BD Biosciences) and incubated in the dark at room temperature for 15 min. Cell apoptosis was analyzed using a Cytoflex flow cytometer (BD Biosciences).

### Western blot

Total protein was extracted from melanocytes using RIPA lysis buffer with 1% protein phosphatase inhibitors. Proteins were separated by 12% SDS-PAGE and transferred to PVDF membranes. Membranes were blocked with 5% non-fat milk for 1 h, incubated with primary antibodies overnight at 4 °C, washed with PBS, and incubated with secondary antibodies at room temperature for 2 h. Signals were detected using the ECL system (Pierce Biotechnology) and analyzed with ImageJ (National Institutes of Health).

### Mfsd4a interference and transfection

The shRNA Mfsd4a vectors and Lipofectamine 3000 (L3000015, Thermo Fisher) were individually diluted in Opti-MEM (31985070, Thermo Fisher). Subsequently, the pre-diluted Lipofectamine 3000 was added to generate transfection complexes. These complexes were then introduced into the melan-a cells. After an incubation period of 4–6 h, the culture medium is replaced. The cells are further incubated for 48–72 h, followed by qPCR analysis to assess the interference efficiency targeting the Mfsd4a gene.

### Luciferase reporter assay

The luciferase reporter vector pmirGLO carrying TYR promoter-WT or TYR promoter-MUT was transfected into melan-a cells. To reveal the binding of MITF to the TYR promoter, pcDNA3.1 or pcDNA3.1-MITF was co-transfected with pmirGLO. After 48 h, luciferase activity was detected using the Luciferase Reporter Assay System (Promega).

### ChIP-qPCR

ChIP assays were performed using the EZ-Magna ChIP Chromatin Immunoprecipitation Kit (17-10086, Sigma-Aldrich) according to the manufacturer’s instructions. In brief, 5 × 10^6 melan-a cells were cross-linked with 1% formaldehyde at room temperature for 10 min. Cells were lysed and sonicated using the Sonics VCX130 (Sonics & Materials) for 10 cycles of 10 s on/20 s off at 50% amplitude. Immunoprecipitation was performed with antibodies against p65 (MA5-15160, Thermo Fisher) and rabbit IgG (31194, Thermo Fisher). qPCR was used to detect the precipitates.

### Immunohistochemistry (IHC)

Embedded tissue sections were deparaffinized with xylene, rehydrated with gradient ethanol, and immersed in 3% hydrogen peroxide at room temperature for 10 min to quench endogenous peroxidase activity. Antigen retrieval was performed in Tris–EDTA buffer (pH 9.0) at 100 °C for 30 min. Sections were incubated with primary antibodies overnight at 4 °C, washed with PBS, incubated with secondary antibodies at room temperature for 30 min, and stained for observation under a microscope.

### Masson-Fontana and HE staining

Paraffin-embedded skin sections (5 μm) were deparaffinized and washed with distilled water. Sections were incubated with Fontana ammonia silver solution in the dark for 16 h, washed 5 times with distilled water, incubated with hyposulfite solution for 5 min, washed again, dehydrated with ethanol, cleared with xylene, and mounted. Melanocytes in the basal layer were counted under a microscope. For HE staining, tissue sections were deparaffinized, baked, stained with hematoxylin and eosin, dehydrated, cleared, and mounted. Observations were made under a microscope.

### Statistical analysis

Data were analyzed using SPSS 24.0 software and expressed as mean ± standard deviation. T-tests were used for comparisons between groups, and *P* < 0.05 was considered statistically significant. Graphs were drawn using GraphPad Prism 8.0.

## Results

### Filiform fire needling therapy alleviates lesions in vitiligo patients

**Fig. 1:**
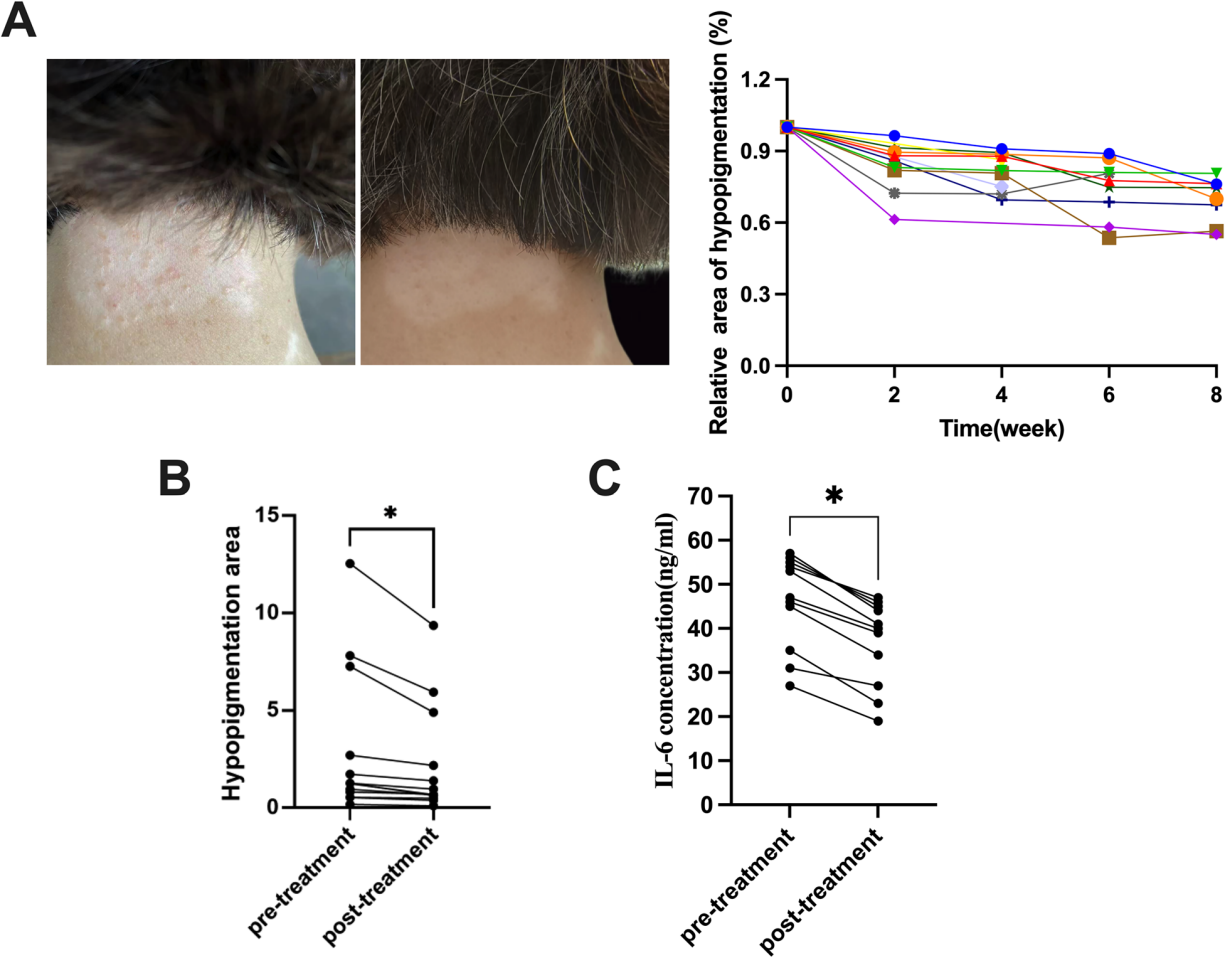
The Efficacy of Filiform Fire Needling Therapy in Enrolled Vitiligo Patients. **A** Filiform fire needle treatment alleviated hypopigmentation in vitiligo patients. **B** Changes of hypopigmentation area in patients. **C** IL-6 levels in the peripheral blood samples decreased after treatment. *P < 0.05

We conducted an 8-week treatment with filiform fire needles on the pathological areas of vitiligo patients (Table 1). As shown in Fig. 1A, B, the degree of depigmentation in patients after treating with filiform fire needles significantly decreased compared with their baseline conditions. After four sessions of filiform fire needle treatment, the lesion areas in all subjects were significantly reduced. It performed a sustained efficacy during the treatment period and the end of the treatment course. The lesions areas have been improved significantly compared to the baseline status (*P* < 0.05). Most of them were improved by initial treatment with a continuous relief obviously. There were 6 patients defined as treatment-related adverse events with grade 1 according to the CTCAE5.0. One case developed to trichrome vitiligo around the skin lesions during treatment, but no depigmentation or progression was observed after a follow-up 3 months. We collected samples from the affected areas of the patients and measured IL-6 using ELISA. The results showed a significant decrease in IL6 levels after filiform fire needle treatment in peripheral blood samples (Fig. 1C).

**Table 1:** Baseline characteristics of enrolled subjects

Variables	n = 11
Gender(male, %)	5(45.45%)
Age(year)	38.36 ± 10.55
Course of disease(year)	3.00(2.00–7.50)
Risk factor(n,%)	
Vaccination	2(18.18%)
Surgery	1(9.09%)
Pregnancy	1(9.09%)
Blood donation	1(9.09%)
Unknown	6(54.55%)
Type(n,%)	
Acrofacial	4(36.36%)
Sporadic	5(45.45%)
Focal	2(18.18%)
History(n,%)	
Colon cancer	1(9.09%)
Hypertension	2(18.18%)
Thyroid disease	2(18.18%)
Alopecia areata	1(9.09%)
None	6(54.55%)
Treatment history(n,%)	
Oral corticosteroids	1(9.09%)
Oral administration of Chinese Medicine	4(36.36%)
Topical glucocorticoids	5(45.45%)
Topical calcineurin inhibitors	6(54.55%)
Topical crisaborole	1(9.09%)
Topical benvitimod	1(9.09%)
NB-UVB	2(18.18%)
308 nm excimer laser	3(27.27%)

### Protective effects of filiform fire needling therapy in monobenzone-induced vitiligo model

**Fig. 2:**
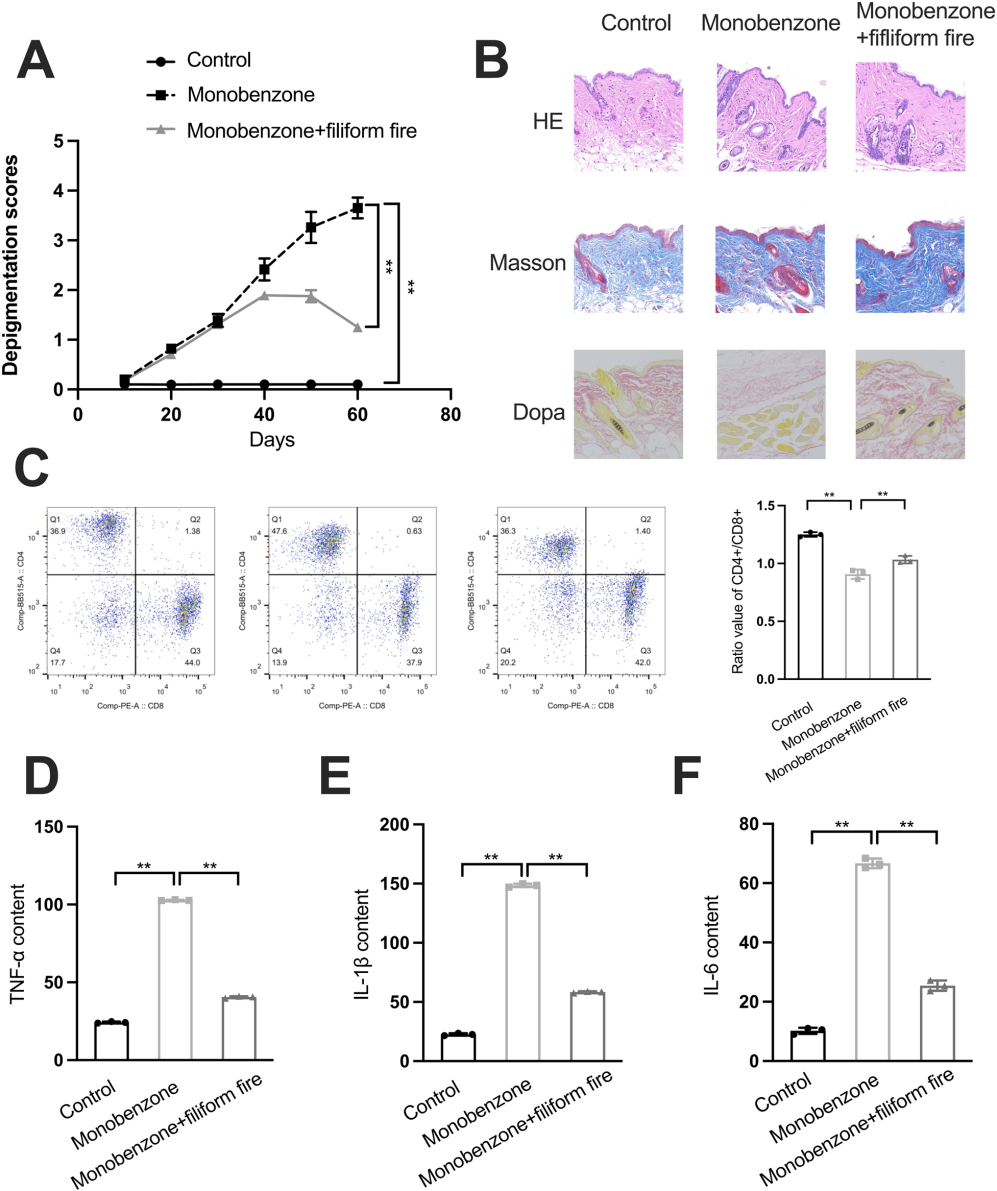
Protective Effects of Filiform Fire Needling Therapy in Monobenzone-Induced Vitiligo Model. **A** Depigmentation scores of the monobenzone-induced vitiligo model and filiform fire needle treatment groups. **B** HE, Masson-Fontana and Dopa staining of the skin tissues in the control, monobenzone-induced vitiligo model and filiform fire needle treatment groups. **C** Flow cytometry analysis of T cell numbers in the vitiligo model and filiform fire needle treatment groups. **D** ELISA analysis of TNF-α levels in the vitiligo model and filiform fire needle treatment groups. **E** ELISA analysis of IL-1β levels in the vitiligo model and filiform fire needle treatment groups. **F** ELISA analysis of IL-6 levels in the vitiligo model and filiform fire needle treatment groups. **P < 0.01

We established a vitiligo model in mice using monobenzone and treated the model with filiform fire needle. We observed the degree of repigmentation in the skin of vitiligo mice before and after filiform fire needle treatment. The depigmentation score significantly increased after administrated with monobenzone, while it obviously decreased when treat with the monobenzone and filiform fire needle (Fig. 2A). HE staining showed that monobenzone treatment led to epidermal thickening and leukocyte infiltration, which were alleviated after filiform fire needle treatment (Fig. 2B). Masson-Fontana staining revealed a significant reduction in melanin content in the models treated with monobenzone, while it was increased by filiform fire needle treatment (Fig. 2B). Flow cytometry analysis indicated an increase in CD8 + T cells and a decrease in the CD4 + /CD8 + T cell ratio when treated with monobenzone, while these effects were reversed by filiform fire needle treatment (Fig. 2C). ELISA assays showed that monobenzone treatment increased the levels of TNF-α, IL-1β, and IL-6, whereas filiform fire needle treatment reduced these levels (Fig. 2D–F).

### Filiform fire needling therapy relieves T cells-mediated melanocytes apoptosis and dysfunction

**Fig. 3:**
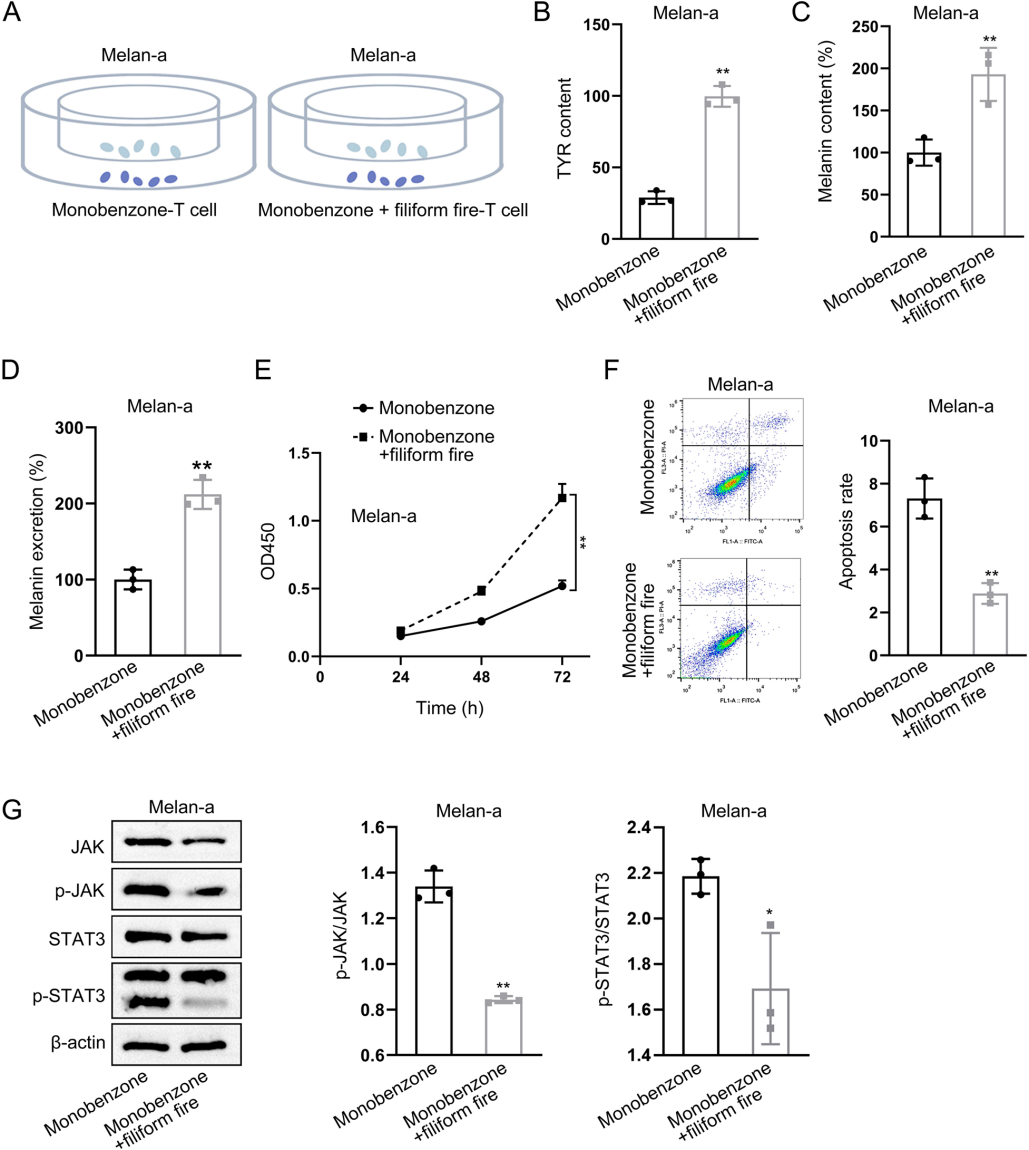
Filiform Fire Needling Therapy Relieves T Cells-mediated Melanocytes Apoptosis and Dysfunction. **A** Co-culture model of T cells isolated from the monobenzone-induced vitiligo model and filiform fire needle treatment groups with melanocytes. **B** ELISA analysis of tyrosinase activity in melanocytes. **C** Measurement of melanin content in melanocytes. **D** Measurement of melanin secretion in melanocytes. **E** CCK8 analysis of melanocyte viability. **F** Flow cytometry analysis of melanocyte apoptosis. **G** Western blot analysis of JAK/STAT3 pathway activity in melanocytes. *P < 0.05; **P < 0.01

T cells were isolated from monobenzone-induced models as well as filiform fire needle treated models and co-cultured with melanocytes in vitro (Fig. 3A). Compared with that co-cultured with the T cells from the model lesions, it demonstrated that the tyrosinase activity in melanocytes was increased significantly after co-culture with T cells from filiform fire needle treated models (Fig. 3B). Moreover, melanin content and secretion levels in melanocytes also increased significantly after co-cultured with T cells from filiform fire needle treated models (Fig. 3C, D). Additionally, co-culture with T cells from filiform fire needle treated lesions increased cell viability and decreased apoptosis of melanocytes after detected by CCK8 assays and flow cytometry analysis, respectively (Fig. 3E, F). Lastly, western blot analysis showed that the pJAK/JAK and pSTAT3/STAT3 ratios in melanocytes decreased significantly when co-cultured with the T cells from filiform fire needle treated lesions (Fig. 3G).

### Filiform fire needling treated lesion T cells regulate melanogenesis-related genes expression and inflammatory cytokines levels

**Fig. 4:**
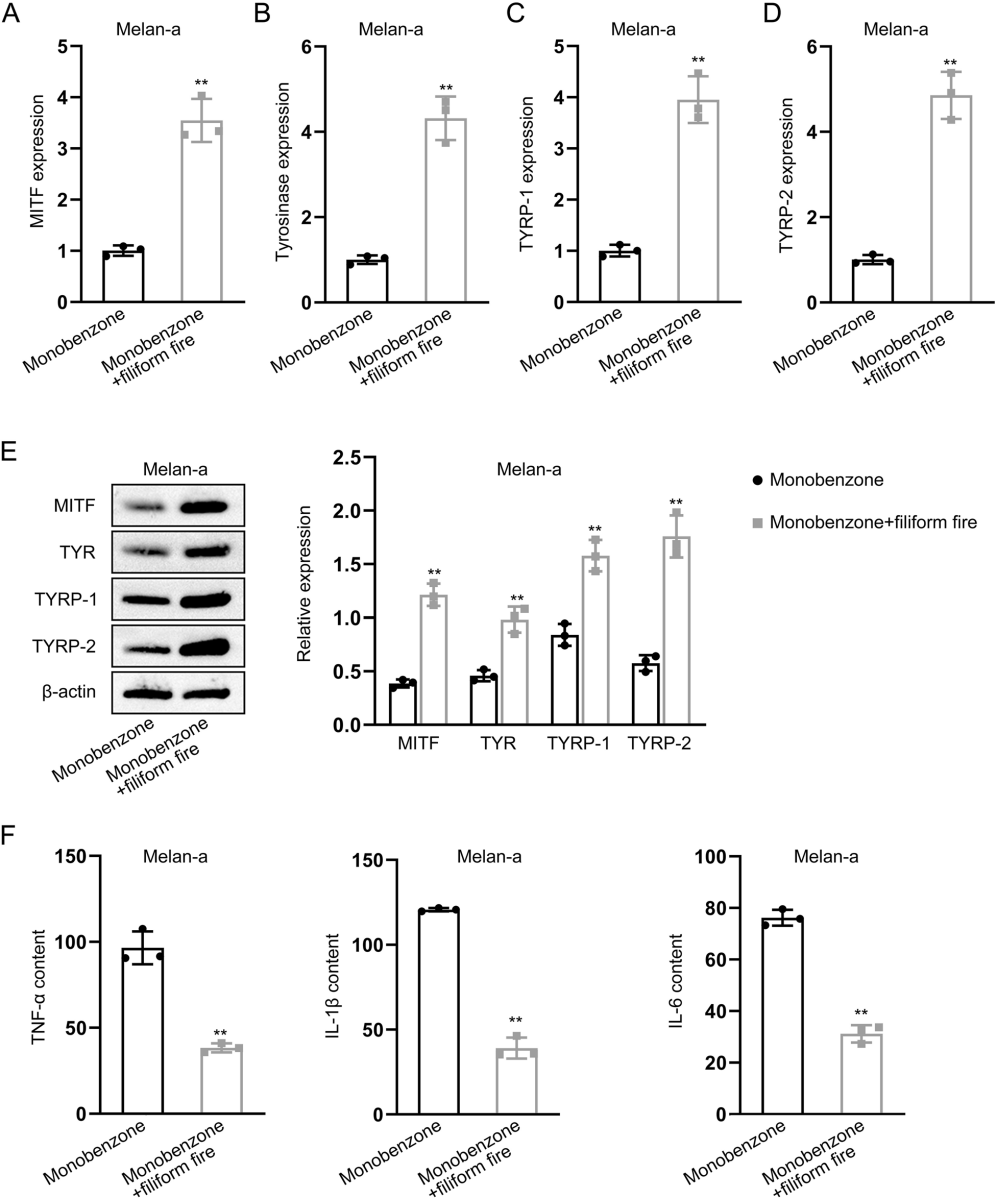
Effects of Filiform Fire Needle on Melanogenesis-Related Genes and Inflammatory Cytokines. **A** qPCR analysis of MITF expression in melanocytes. **B** qPCR analysis of Tyrosinase expression in melanocytes. **C** qPCR analysis of TYRP-1 expression in melanocytes. **D** qPCR analysis of TYRP-2 expression in melanocytes. **E** Western blot analysis of MITF, Tyrosinase, TYRP-1, and TYRP-2 expression in melanocytes. **F** ELISA analysis of inflammatory cytokine expression in melanocytes. **P < 0.01

We analyzed the expression of melanogenesis-related genes using qPCR and western blot. The results showed that the expression of MITF, Tyrosinase, TYRP-1, and TYRP-2 increased after co-cultured with the T cells from monobenzone and filiform fire needle treated models compared to with that from monobenzone-treated lesions (Fig. 4A–E). Additionally, we found that co-cultured with the T cells from co-treated models decreased the levels of TNF-α, IL-1β, and IL-6 in melanocyte according to the results of ELISA assay (Fig. 4F).

### Filiform fire needling therapy inhibits JAK/STAT3 pathway activity through Mfsd4a

**Fig. 5:**
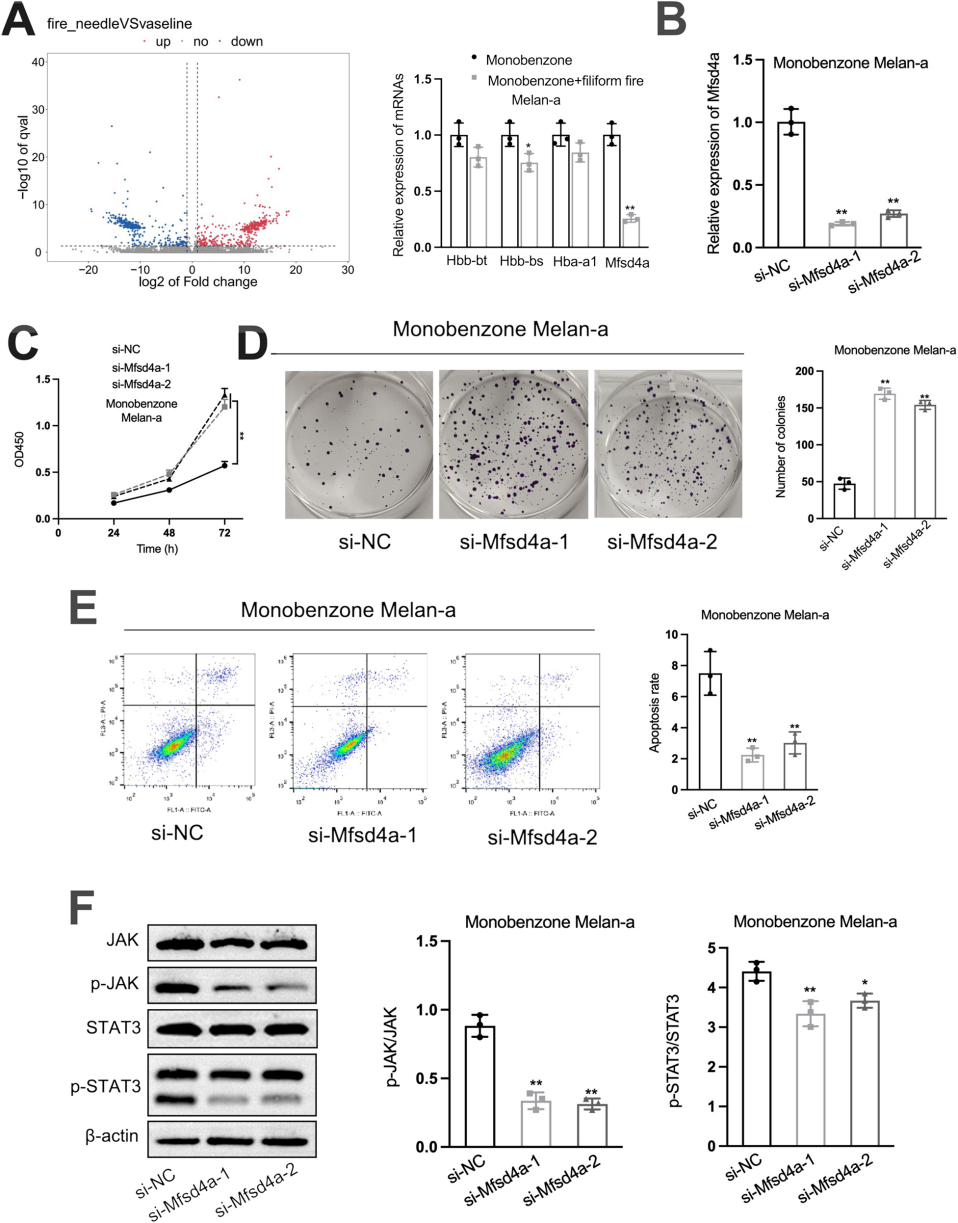
Mfsd4a Promotes Vitiligo Through JAK/STAT3 Pathway Activation. **A** Volcano plot of differentially expressed genes in melan-a cells co-cultured with T cells from filiform fire needle-treated vs. untreated lesions (RNA-seq), with qPCR validation of Hbb-bt, Hbb-bs, Hba-a1, and Mfsd4a. **B** Verification of Mfsd4a knockdown efficiency in melan-a cells co-cultured with T cells from monobenzone-induced vitiligo lesions. **C** CCK8 analysis of Mfsd4a effects on melanocyte viability. **D** Colony formation assay of Mfsd4a effects on melanocyte proliferation. **E** Flow cytometry analysis of Mfsd4a effects on melanocyte apoptosis. **F** Western blot analysis of Mfsd4a effects on JAK/STAT3 pathway activity. *P < 0.05; **P < 0.01

**Fig. 6:**
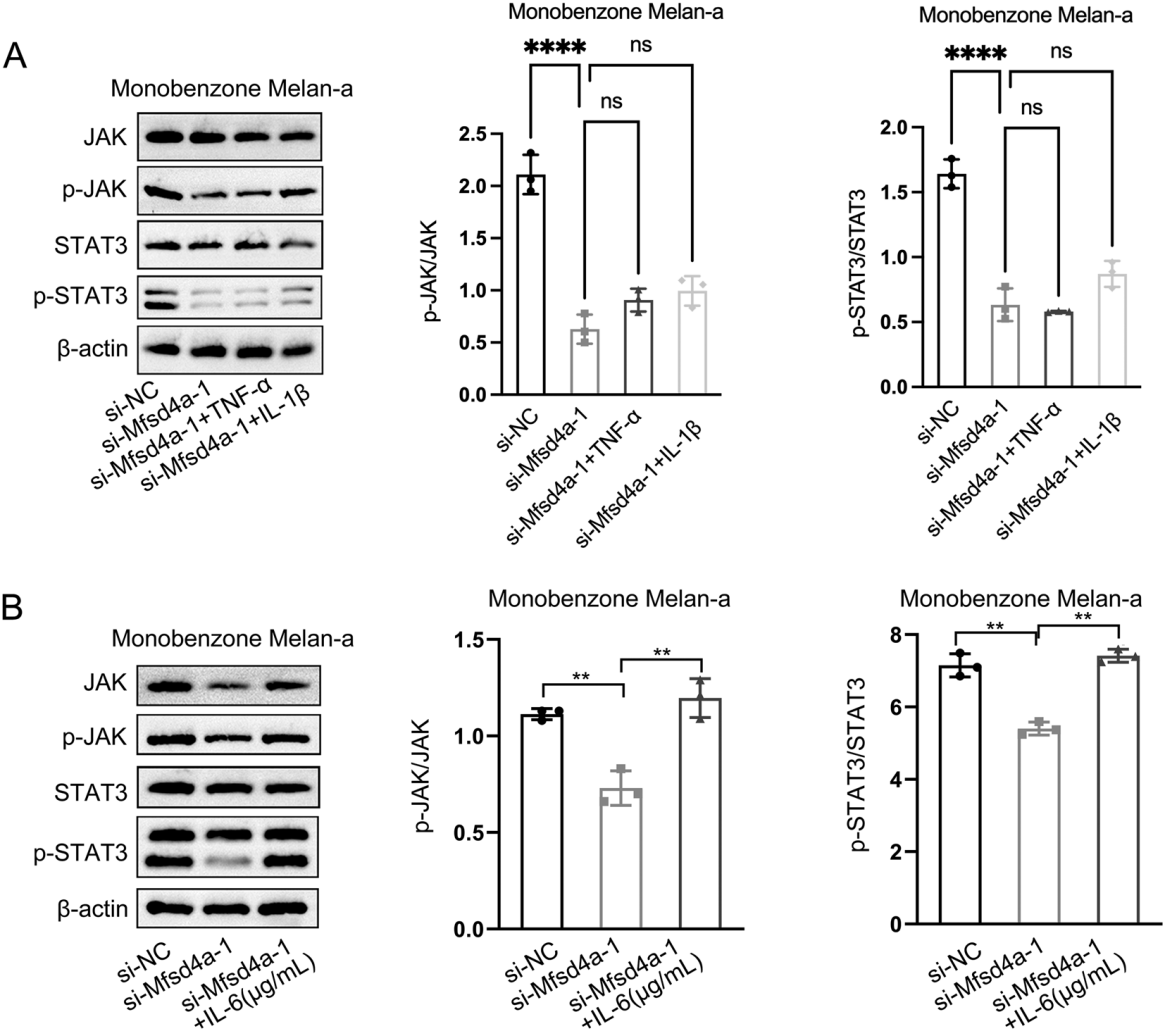
Mfsd4a Activates JAK/STAT3 Pathway Through IL-6. **A** Western blot analysis of JAK/STAT3 pathway activity in Mfsd4a-modulated melanocytes under TNF-α and IL-1β stimulation. **B** Western blot analysis of JAK/STAT3 pathway activity in Mfsd4a-knockdown melanocytes with exogenous IL-6. **P < 0.01; ****P < 0.0001; *ns* no significance

To dig into the underlying molecular mechanism of filiform fire needling therapy-induced T cells in melanocytes, we performed RNA-seq on melanocytes which co-cultured with T cells from monobenzone with or without filiform fire needle treated lesions. According to the results, we identified four candidate genes (Hbb-bt, Hbb-bs, Hba-a1, and Mfsd4a) low-expressed in the melanocytes which co-cultured with T cells form co-treated lesions. Mfsd4a was selected as the underlying molecule according to the results of qPCR assay (Fig. 5A). Mfsd4a interference vectors were transfected to melanocytes and then co-cultured with monobenzone-induced T cells. qPCR assay was utilized to verify transfection efficiency (Fig. 5B). CCK8 and colony formation assays showed that Mfsd4a interference significantly promoted melanocyte proliferation (Fig. 5C, D), and flow cytometry analysis indicated that Mfsd4a interference significantly inhibited melanocyte apoptosis (Fig. 5E). Western blot analysis showed that Mfsd4a interference decreased the pJAK/JAK and pSTAT3/STAT3 ratios in melanocytes (Fig. 5F). Like filiform fire needle treatment, Mfsd4a interference also reduced the levels of inflammatory cytokines in melanocytes treated with monobenzone (Figure S1). Moreover, overexpression of Mfsd4a in normal melanocytes inhibited their activity and melanin production. Co-culture of these melanocytes with T cells from co-treated lesions restored their activity and melanin production (Figure S2). However, when melanocytes with Mfsd4a interference were treated with exogenous TNF-α and IL-1β, these cytokines did not affect the influence of Mfsd4a on JAK/STAT3 pathway activity. Only exogenous IL-6 treatment reversed the inhibitory effect of Mfsd4a interference on JAK/STAT3 pathway activity (Fig. 6A, B). These results suggest that Mfsd4a modulates melanocyte activity through the IL-6/JAK/STAT3 pathway.

### Filiform fire needling therapy relieves T cells-mediated melanocytes dysfunction through Mfsd4a/MITF

**Fig. 7:**
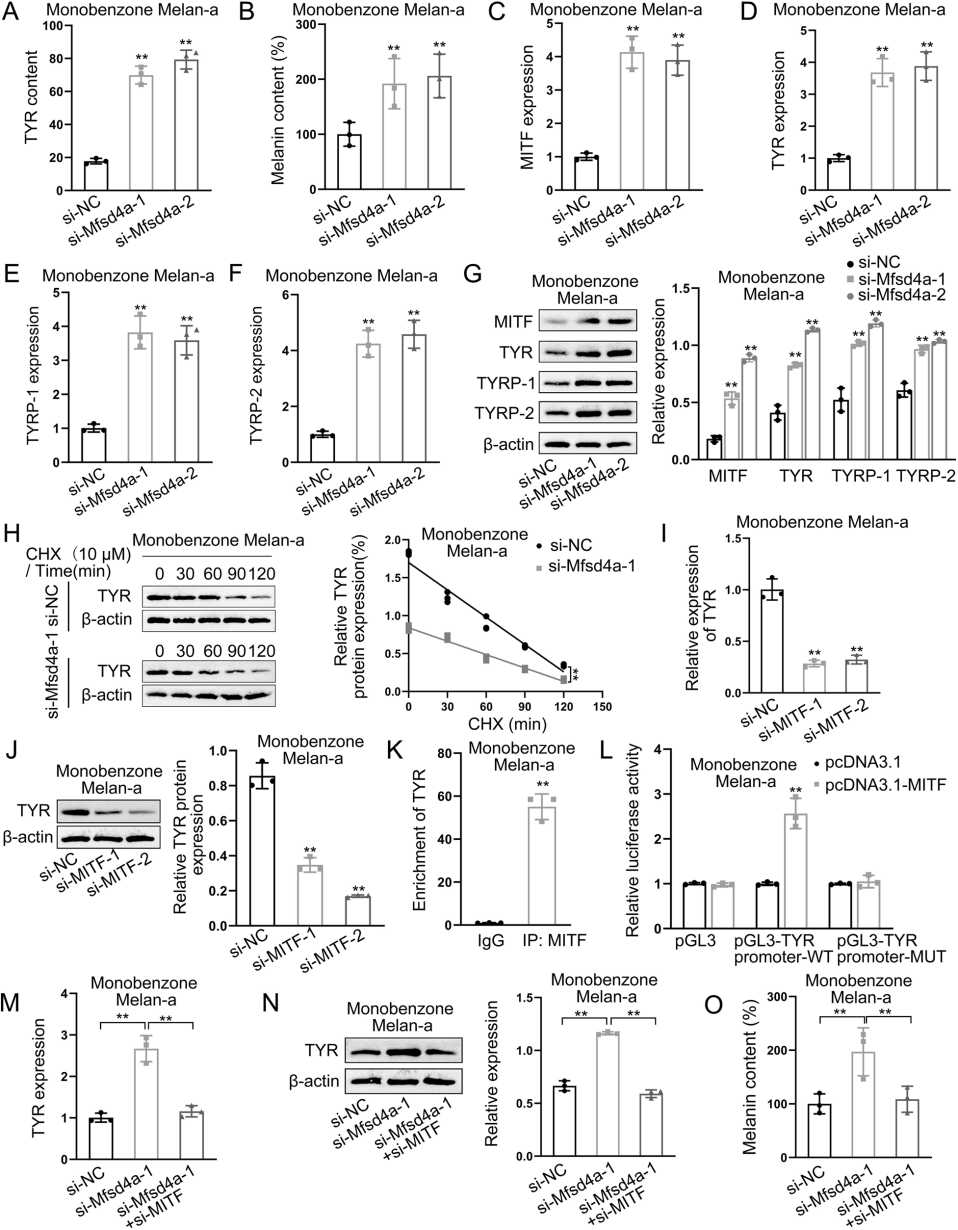
Effects of Mfsd4a on Melanin Production. **A** ELISA analysis of Mfsd4a interference effects on tyrosinase activity. **B** Measurement of melanin content in melanocytes with Mfsd4a interference. **C** qPCR analysis of MITF expression in melanocytes with Mfsd4a interference. **D** qPCR analysis of Tyrosinase expression in melanocytes with Mfsd4a interference. **E** qPCR analysis of TYRP-1 expression in melanocytes with Mfsd4a interference. **F** qPCR analysis of TYRP-2 expression in melanocytes with Mfsd4a interference. **G** Western blot analysis of MITF, Tyrosinase, TYRP-1, and TYRP-2 expression in melanocytes with Mfsd4a interference. **H** Western blot analysis of Mfsd4a effects on TYR stability. **I** qPCR analysis of MITF interference effects on TYR expression in melanocytes. **J** Western blot analysis of MITF interference effects on TYR expression in melanocytes. **K** ChIP-qPCR analysis of MITF binding to TYR promoter. **L** Luciferase reporter assay analysis of MITF transcriptional activation of TYR. **M** qPCR verification that Mfsd4a interference activates TYR through MITF transcription. **N** Western blot verification that Mfsd4a interference activates TYR through MITF transcription. **O** Analysis of Mfsd4a effects on melanin content through MITF. **P < 0.01

To determine whether filiform fire needling therapy affects melanogenesis through Mfsd4a, we measured tyrosinase activity and melanin content in melanocytes. Mfsd4a interference significantly increased tyrosinase activity and melanin content (Fig. 7A, B). qPCR and Western blot analyses showed that Mfsd4a interference significantly increased the expression of MITF, Tyrosinase, TYRP-1, and TYRP-2 (Fig. 7C–G). Previous studies have shown that MITF regulates the expression of all melanogenesis-related proteins [13, 14]. We hypothesized that Mfsd4a affects TYR stability through MITF. qPCR and Western blot analyses showed that MITF interference decreased TYR mRNA and protein expression (Fig. 7I, J). ChIP-qPCR and luciferase reporter assays demonstrated that MITF binds to and activates the TYR promoter (Fig. 7K, L). Rescue experiments showed that MITF interference reversed the promoting effect of Mfsd4a interference on TYR mRNA and protein expression, as well as melanin content (Fig. 7M–O).

## Discussion

**Fig. 8:**
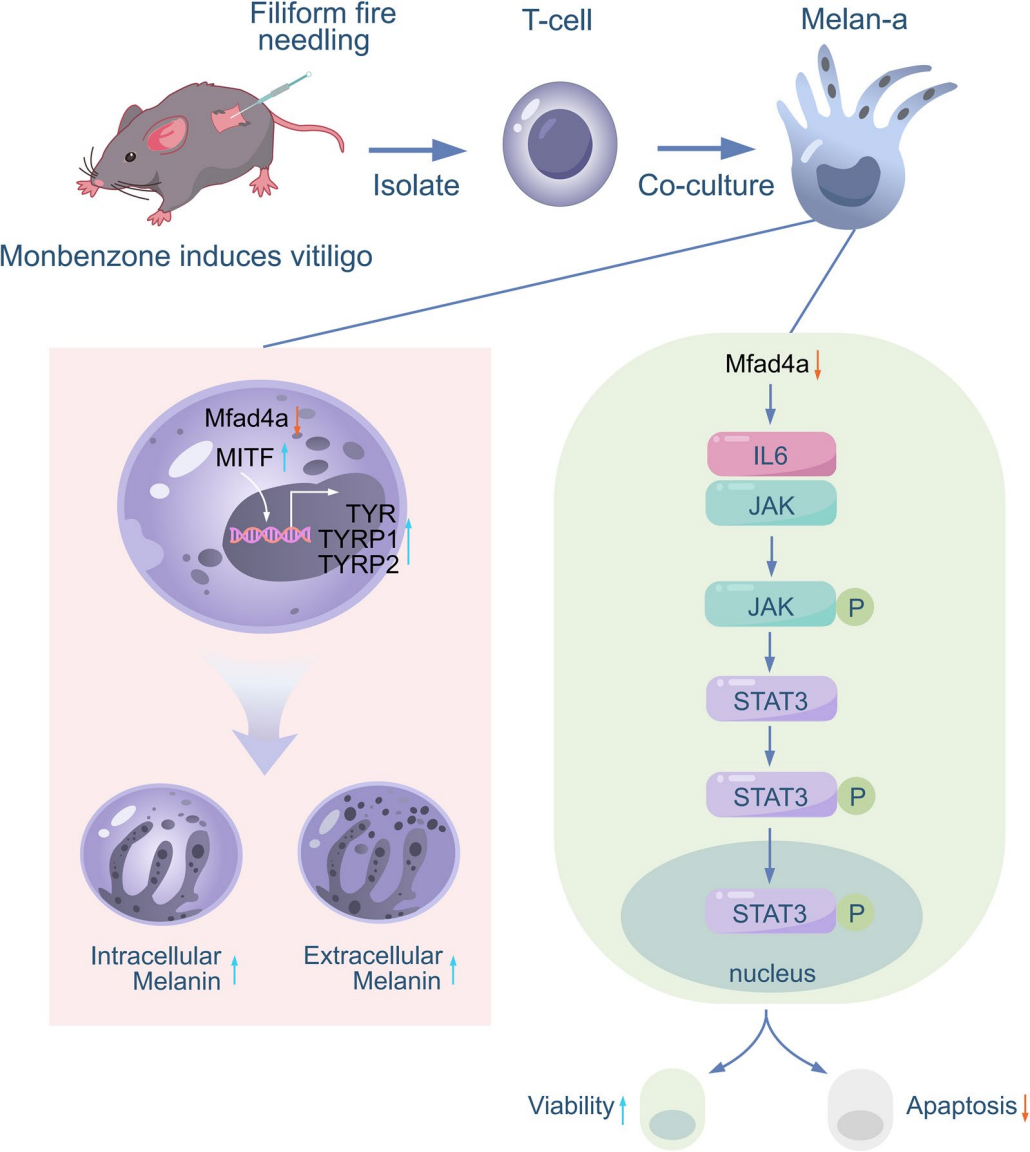
Schematic diagram of filiform fire needling therapy relieves T cells-mediated melanocyte apoptosis and dysfunction by inhibiting JAK/STAT3 pathway via Mfsd4a in vitiligo

Present study uncovered a novel mechanism of filiform fire needling therapy for vitiligo. Filiform fire needling therapy alleviates T cells-mediated melanocyte apoptosis and dysfunction by inhibiting JAK/STAT3 pathway via Mfsd4a. Vitiligo is a progressive depigmenting skin disease characterized by depigmented white patches caused by the inhibition of the tyrosinase system, melanocyte loss, and activation of CD8 + T lymphocytes [4, 15]. Filiform fire needling therapy has been demonstrated as an effective and safety option for vitiligo [11]. In current study, we observed significant improvement in skin depigmentation after filiform fire needle treatment in patients with vitiligo with an outstanding safety. Additionally, we found that the IL-6 levels were decreased after treated with filiform fire needle. In vitiligo mice model, filiform fire needle treatment also effectively alleviated skin depigmentation and increased count of melanocytes in hair follicles. The infiltration of lymphocytes was also decreased after received filiform fire needling therapy in vivo (Fig. 8).

Previous studies have shown that the loss of melanocytes is related to the activation of CD8 + T lymphocytes [16]. Acupuncture has the effect on stimulating meridians, regulating visceral functions, promoting local blood circulation, increasing local nutrient supply, stimulating the tyrosinase system, and increasing melanocyte activity. Additionally, acupuncture has been determined to regulate the immune system with minimal toxicity [11, 17], highlighting its advantages in treating vitiligo. In this study, we found that CD8 expression significantly decreased in the damaged tissues of models treated with filiform fire needle, suggesting that the treatment inhibited the differentiation of lymphocytes to CD8 + T cells, contributing to protect melanocytes. Our results confirmed that CD8 + T cell numbers increased significantly, and melanocyte numbers decreased in the vitiligo model, whereas filiform fire needle treatment inhibited CD8 + T cell numbers and increased melanocyte numbers. Furthermore, since acupuncture regulates the tyrosinase system, and previous studies have found that IFN-γ secreted by CD8 + T cells induced chemokine production by activating the JAK/STAT3 pathway, promoting melanocyte apoptosis [4], and JAK/STAT3 pathway has been uncovered as a target for dermatological diseases treatment[18]. Notably, JAK/STAT3 pathway activity was inhibited in the tissues of mice treated with filiform fire needle.

In the pathogenesis of vitiligo, the accumulation of natural killer (NK) cells and the production of variety inflammatory proteins and cytokines also contribute to disease development [19]. For example, Xie et al. found that IFN-γ produced by T lymphocytes near vitiligo skin lesions promotes HSP70i expression [20], and Peng et al. found elevated levels of IL-8, IL-6, TNF-α, IFN-γ, and IL-1β in vitiligo mouse skin [4]. We isolated T lymphocytes from the vitiligo mouse model and co-cultured them with melanocytes. It was demonstrated that T cells from filiform fire needle treated lesion significantly increased melanocyte activity and inhibited melanocyte apoptosis. Our research also found the levels of inflammatory cytokines such as TNF-α, IL-1β, and IL-6 were decreased after co-cultured with T cells from filiform fire needle treated lesion.

To elucidate the underlying mechanism of filiform fire needle in treating vitiligo, we performed RNA-seq analysis on co-cultured melanocytes and identified significant changes in Mfsd4a. Interfering with Mfsd4a in melanocytes co-cultured with vitiligo model T cells significantly improved melanocyte activity, inhibited the levels of inflammatory cytokines TNF-α, IL-1β, and IL-6, reduced melanocyte apoptosis, and inhibited JAK/STAT3 pathway activation. The protein encoded by Mfsd4a belongs to the membrane protein family. It is crucial for the development of the blood–brain barrier and participates in lipid such as polyunsaturated fatty acids transport, influencing the function of nerve cells. Mfsd4a may also play a role in the immune system, participating in regulating the function and activity of immune cells, and its abnormal expression may be related to certain immune-related diseases. Interestingly, when we interfered with Mfsd4a in melanocytes and treated them with exogenous inflammatory cytokines, only administrating with exogenous IL-6 reversed the inhibitory effect of Mfsd4a interference on the JAK/STAT3 signaling pathway, indicating that filiform fire needle treats vitiligo by inhibiting Mfsd4a expression through inhibiting IL6, thereby decreasing JAK/STAT3 signaling pathway activity and regulating melanocyte viability. 

Melanocytes function to synthesize the pigment melanin and transfer it to keratinocytes, playing a significant role in skin pigmentation disorders [21, 22]. Our study found that after co-cultured with filiform fire needle-induced T cells, the melanin content and secretion were restored in melanocytes, indicating that filiform fire needle therapy not only protects T cells-mediated melanocytes injury but also relieved T cells-induced melanocytes dysfunction. Previous studies have shown that mature melanocyte melanin synthesis occurs in melanosomes. Melanosomes are unique organelles in melanocytes that contain key enzymes regulating pigment production, such as tyrosinase (TYR), tyrosinase-related protein-1 (TYRP-1), and tyrosinase-related protein-2 (TYRP-2) [23, 24]. Our research also found that the content of TYR, TYRP1, and TYRP2 increased in the skin tissues and melanocytes treated with filiform fire needle or filiform fire needle-induced T cells. Moreover, Mfsd4a significantly influenced the content of TYR, TYRP1, and TYRP2. Previous studies have shown that the activation of the transcription factor MITF is crucial for the upregulation of TYR, TYRP1, and TYRP2 [14], and MITF expression is also inhibited by Mfsd4a. Our study confirmed that MITF transcriptionally activates TYR. Although we did not further investigate the molecular mechanism between Mfsd4a and MITF, previous studies have suggested that the WNT, NF-kB, and JNK pathways contributed to regulate MITF expression and activity [25, 26]. Notably, Choi et al. proposed that IL-4 downregulates MITF through the JAK2/STAT6 signaling pathway [27], and Swope et al. proposed that IL-6 inhibits melanogenesis [28]. Based on this, we hypothesize that Mfsd4a regulates MITF activity through IL-6-mediated JAK/STAT3 pathway activation, ultimately affecting melanin production.

## Supplementary Information

Supplementary file 1.

Supplementary file 2.

Supplementary file 3.

Supplementary file 4.

Supplementary file 5.

Supplementary file 6.

Supplementary file 7.

Supplementary file 8.

Supplementary file 9.

Supplementary file 10.

Supplementary file 11.

Supplementary file 12.

Supplementary file 13.

## Data Availability

The data supporting this study’s findings are available from the author Yue Shi upon reasonable request.

## Abbreviations

ATCC: American Type Culture Collection
BSA: Body Surface Area
CCK8: Cell Counting Kit-8
ChIP: Chromatin Immunoprecipitation
cDNA: Complementary DNA
CTCAE: Common Terminology Criteria for Adverse Events
ELISA: Enzyme-Linked Immunosorbent Assay
FFN: Filiform Fire Needling (Therapy)
HE: Hematoxylin and Eosin
IFN-γ: Interferon Gamma
IgG: Immunoglobulin G
IHC: Immunohistochemistry
IL-1β: Interleukin-1 Beta
IL-6: Interleukin-6
JAK: Janus Kinase
MITF: Microphthalmia-associated Transcription Factor
Mfsd4a: Major Facilitator Superfamily Domain Containing 4A
NK: Natural Killer (cells)
PBS: Phosphate-Buffered Saline
PVDF: Polyvinylidene Fluoride
qPCR: Quantitative Polymerase Chain Reaction
RIPA: Radioimmunoprecipitation Assay (buffer)
RNA-seq: RNA Sequencing
RT-qPCR: Reverse Transcription Quantitative PCR
SDS-PAGE: Sodium Dodecyl Sulfate-Polyacrylamide Gel Electrophoresis
shRNA: Short Hairpin RNA
siRNA: Small Interfering RNA
STAT3: Signal Transducer and Activator of Transcription 3
TNF-α: Tumor Necrosis Factor Alpha
TYR: Tyrosinase
TYRP-1: Tyrosinase-Related Protein-1
TYRP-2: Tyrosinase-Related Protein-2
