# Supplementary figures and images for: Filiform fire needling therapy relieves T cells-mediated melanocyte apoptosis and dysfunction by inhibiting JAK/STAT3 pathway via Mfsd4a in vitiligo
Source: Chin Med. 2025 Jul 24;20:117. doi: 10.1186/s13020-025-01172-4 (PMC12288293; doi:10.1186/s13020-025-01172-4)

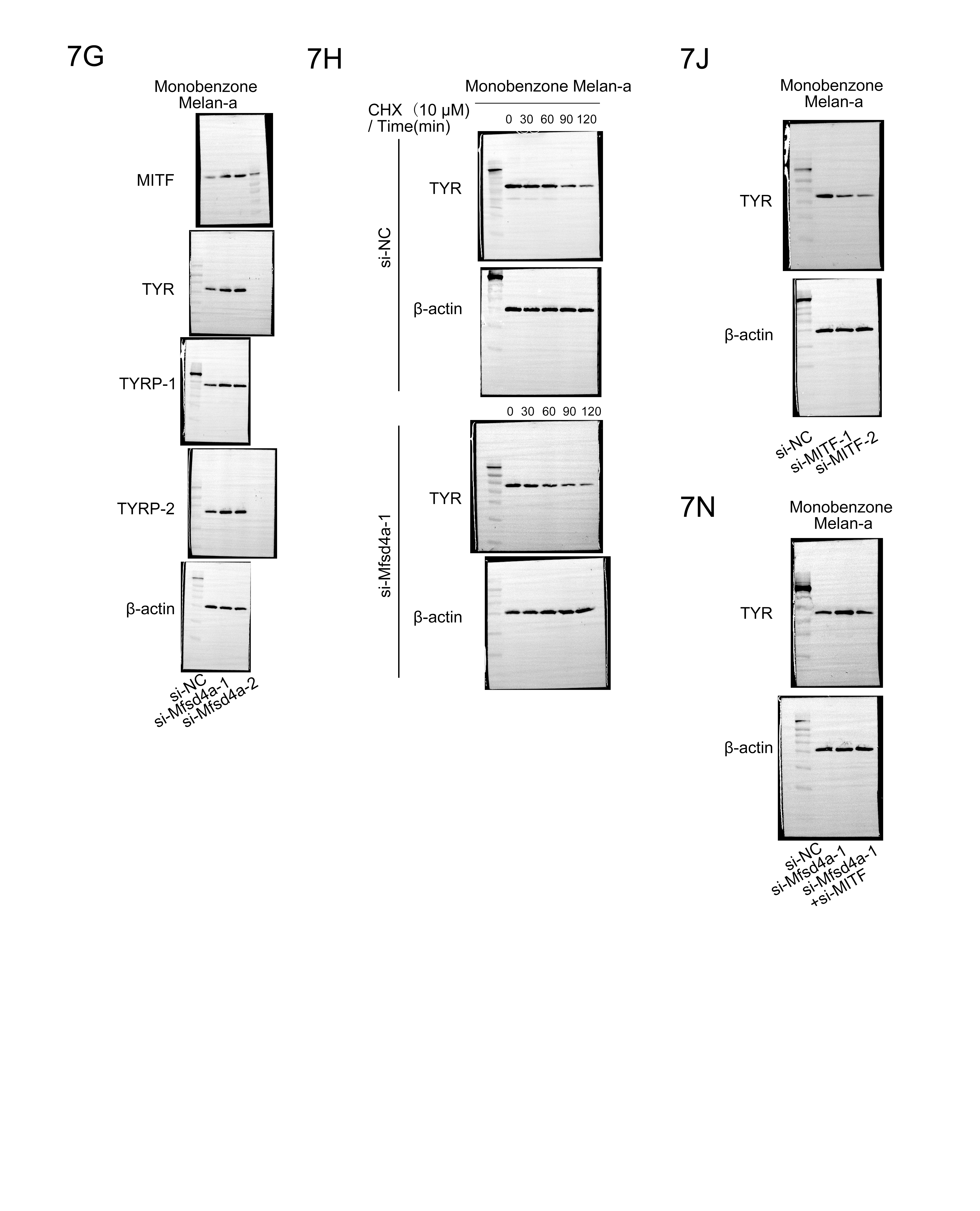

Supplement: Supplementary file 1 [file 13020_2025_1172_MOESM1_ESM.tif]

A

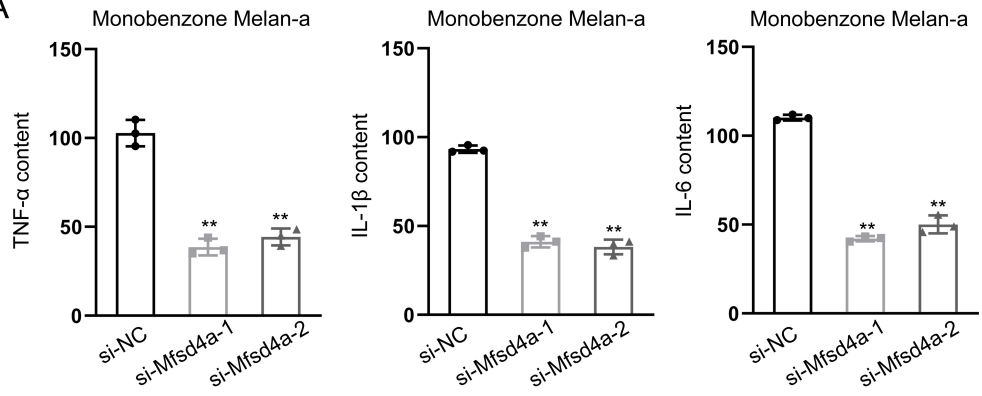

Supplementary Figure 1. Mfsd4a Promotes Inflammatory Cytokine Expression \*\* $P < 0.01$

Supplement: Supplementary file 3 [file 13020_2025_1172_MOESM3_ESM.pdf]

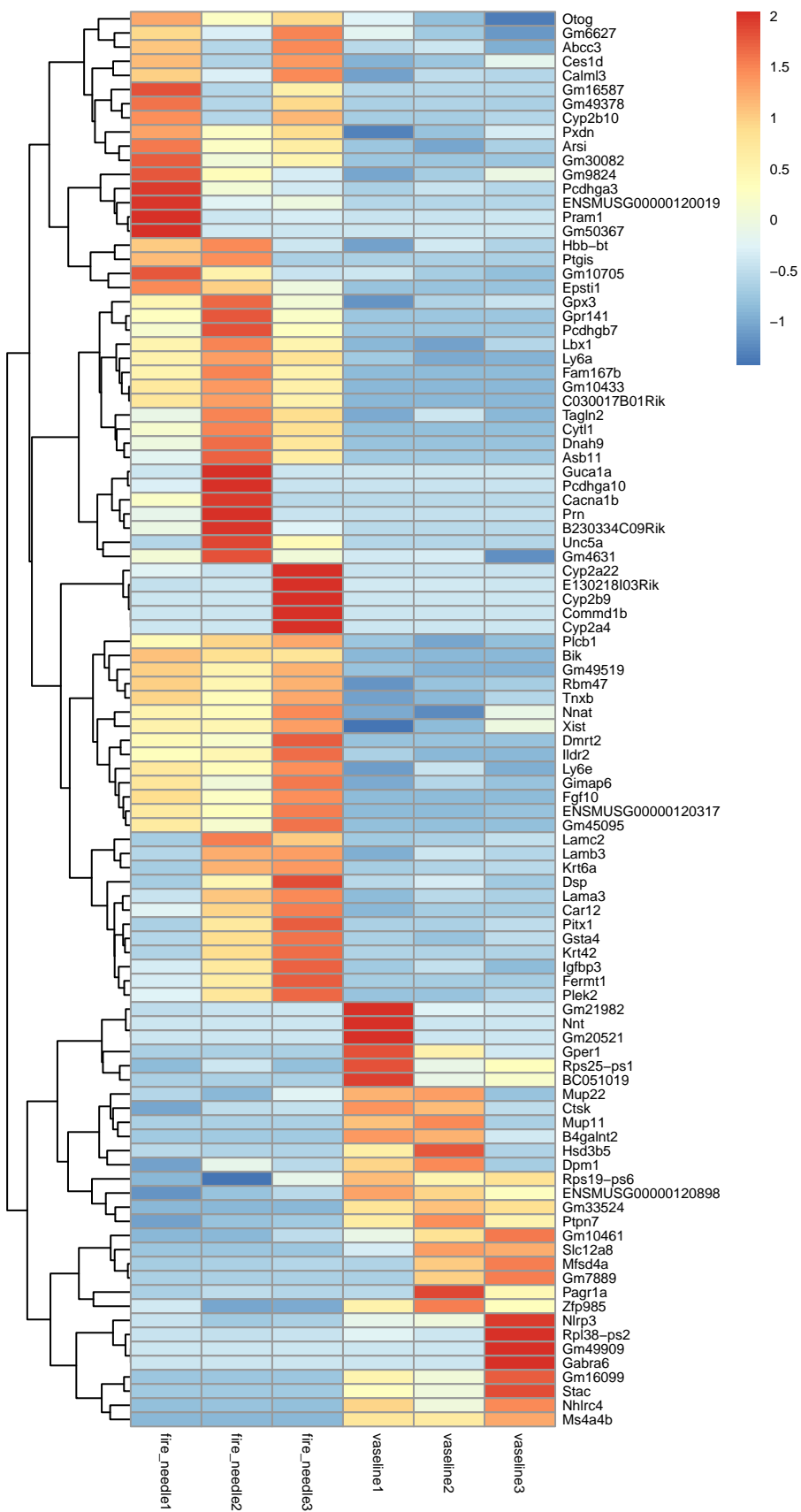

Supplement: Supplementary file 12 [file 13020_2025_1172_MOESM12_ESM.pdf]

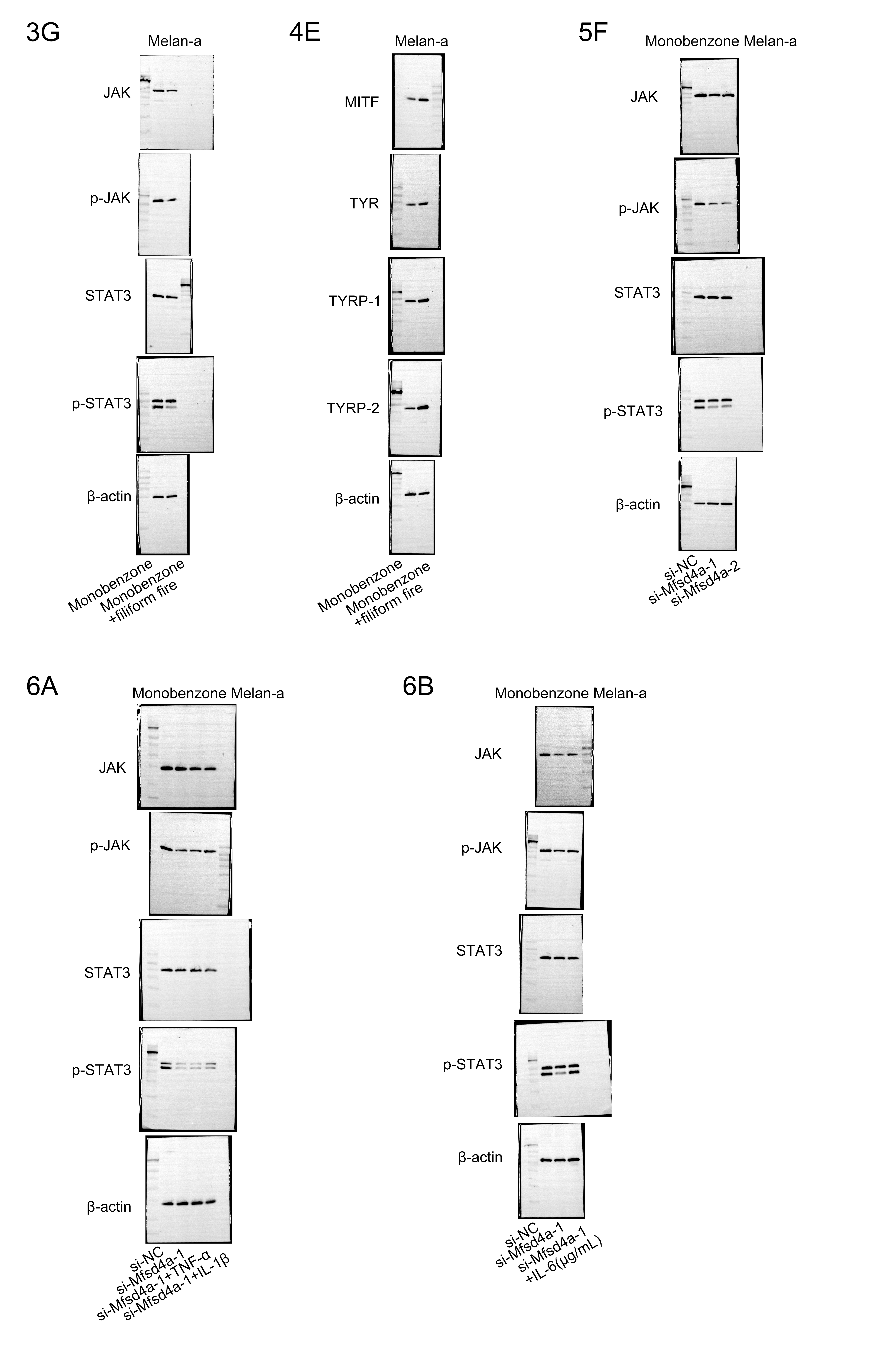

Supplement: Supplementary file 13 [file 13020_2025_1172_MOESM13_ESM.tif]
