# Supplementary material for: Filiform fire needling therapy relieves T cells-mediated melanocyte apoptosis and dysfunction by inhibiting JAK/STAT3 pathway via Mfsd4a in vitiligo
Source: Chin Med. 2025 Jul 24;20:117. doi: 10.1186/s13020-025-01172-4 (PMC12288293; doi:10.1186/s13020-025-01172-4)
Supplement: Supplementary file 4 [file 13020_2025_1172_MOESM4_ESM.pdf]

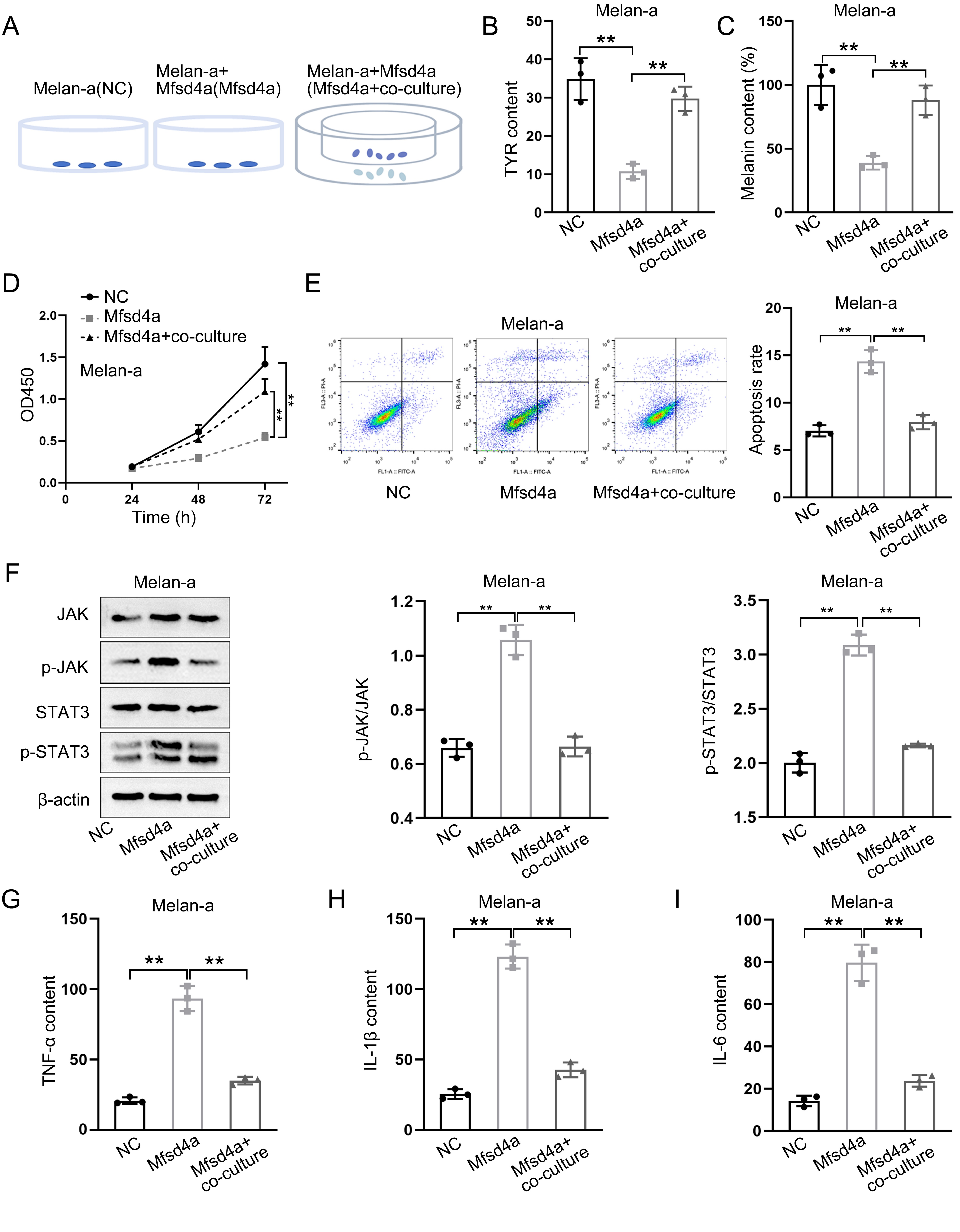

Supplementary Figure 2. Filiform Fire Needle-induced T Cell from Lesions Promotes Melanocyte Activity and Melanin Production Through Mfsd4a

(A)Co-culture model. (B) Measurement of TYR content. (C) Measurement of melanin content. (D) Analysis of melanocyte viability. (E) Measurement of melanocyte apoptosis. (F) Analysis of JAK/STAT3 pathway activity. (G-I) Measurement of inflammatory cytokine content. ***P*<0.01
